# Towards Alignment Independent Quantitative Assessment of Homology Detection

**DOI:** 10.1371/journal.pone.0000113

**Published:** 2006-12-27

**Authors:** Avihay Apatoff, Eddo Kim, Yossef Kliger

**Affiliations:** 1 Compugen Ltd, Tel Aviv, Israel; 2 The Mina and Everard Goodman Faculty of Life Sciences, Bar-Ilan University, Ramat Gan, Israel; Wellcome Trust Centre for Human Genetics, United Kingdom

## Abstract

Identification of homologous proteins provides a basis for protein annotation. Sequence alignment tools reliably identify homologs sharing high sequence similarity. However, identification of homologs that share low sequence similarity remains a challenge. Lowering the cutoff value could enable the identification of diverged homologs, but also introduces numerous false hits. Methods are being continuously developed to minimize this problem. Estimation of the fraction of homologs in a set of protein alignments can help in the assessment and development of such methods, and provides the users with intuitive quantitative assessment of protein alignment results. Herein, we present a computational approach that estimates the amount of homologs in a set of protein pairs. The method requires a prevalent and detectable protein feature that is conserved between homologs. By analyzing the feature prevalence in a set of pairwise protein alignments, the method can estimate the number of homolog pairs in the set independently of the alignments' quality. Using the HomoloGene database as a standard of truth, we implemented this approach in a proteome-wide analysis. The results revealed that this approach, which is independent of the alignments themselves, works well for estimating the number of homologous proteins in a wide range of homology values. In summary, the presented method can accompany homology searches and method development, provides validation to search results, and allows tuning of tools and methods.

## Introduction

Homology detection is a key step in predicting the function of newly discovered proteins. Different methods for homology detection are currently available, and can be divided into sequence-based and structure-based methods. Sequence-based methods rely on estimated evolutionary models that aim at reconstructing the evolutionary courses that relate the protein sequences. The structure-based approach uses protein structure data and allows searching for similar proteins over a structure classification database using structure alignment methods.

An elementary biological dogma states that the 3D structure of a protein determines its function. Data of protein structure is therefore a superior representation of proteins over sequence data. While sequence alignment works best for closely-related homologous proteins, i.e. proteins sharing at least 40% of their amino acid residues, structure alignment produces a significant alignment even between highly diverged homologous proteins [1]. Public databases offer classifications of protein structures [2], e.g. SCOP database, which contains manually inspected structures that are classified in a hierarchical manner [3]. Several methods for searching against such databases were developed [4]–[7], and may detect homologous proteins unrecoverable in regular sequence-based searches. However, although the amount of solved protein structures grows rapidly, it lags behind sequence data. To date, the protein data bank [8] holds structure data of less than 40,000 proteins and protein fragments, while Swiss-Prot knowledgebase holds above 200,000 sequences [9].

High level of sequence conservation is a strong indication for homology. Therefore, sequence alignment methods are frequently used when searching for homologous proteins. These methods use different heuristic algorithms for maximizing the alignment, return a score per alignment of two proteins, and the statistical significance of the pairing [10], [11]. Using such methods to align closely-related homologous proteins often produces a significant long sequence alignment. For distantly-related proteins, however, both the sequence similarity and the alignment length are often reduced, resulting in a decreased statistical significance of the alignment. Moreover, distantly-related homologs might not be included in search results due to stringent default parameters. In such cases, lowering the cutoff value could allow the extraction of distant homologs, but would also amplify the background noise of randomly paired proteins.

Advanced approaches, which are being continuously developed, offer improved homology detection and a reduced background noise in alignment results [12]–[15]. Nevertheless, there is no single superior solution that fits for all homology analyses. Different protein alignment tools, and different configurations of those tools, provide varying results for the same query and subject protein sets. An external independent method that will allow assessment and comparison of homology detection techniques is required.

Herein, we present a computational method for estimating the amount of homologous proteins in a set of protein pairs – the *Fhom Estimator* (Fraction of HOMologs Estimator). The method performs a probabilistic analysis for a set of protein pairs and estimates the fraction of homologs in it. Previously, the method was used for guiding the development of a new scoring function that improved identification of novel viral piracy events [16]. In the current study, we established the reliability of the method using a well-accepted standard of truth dataset of homologous proteins, and demonstrated that the method has a wide dynamic range, that goes down to as low as 0.01% of homologs. Most importantly, we confirmed that our method neither depends on the alignment itself, nor on the level of sequence identity. This approach allows quality assessment of homology search results.

## Results

### A method to estimate the amount of homologous proteins in a set of protein pairs

We developed a computational method to estimate the amount of homologous proteins in a set of protein pairs (e.g. alignment hits). The estimator requires two protein sets, and a protein feature X, which is prevalent in both sets, and conserved among homologous proteins. Ideally, this feature will always be conserved among homologs, i.e., for a given pair of homologs, if a protein has X, so will its homolog, and if a protein lacks X its homolog will not have X as well. In contrast, not every pair of proteins that possess X are homologs, or else we could infer homology by simply observing the conservation of X. Of course, the ability to reliably detect the feature X is essential. Our method estimates the results of any procedure that aims at pairing homologous proteins from two sets of proteins. The prevalence of the protein feature X enables us to estimate the amount of random pairs in the results. Subtracting the estimated random pairs yields the estimated amount of real pairs of homologs in the results - *Fhom*. The rest of this section describes the method in detail, and illustrates it using a hypothetical example.

Protein pairs can be classified into one of four subgroups according to the presence (or absence) of the feature X in them. Proteins that have the feature X are labeled Y, and proteins that lack this feature are labeled N. Accordingly, protein pairs where both proteins have (or lack) the feature X, correspond to the YY (or NN) subgroup, while protein pairs where one protein has (or lacks) the feature X, but the other protein lacks (or has) it, correspond to the YN (or NY) subgroup.

To exemplify, let us assume a hypothetical set of 1000 aligned protein pairs. Such a set may be the result of pairing the proteins of two sets, a query set and a subject set. Let us define these protein sets to have protein feature X prevalence of 15% and 20%, respectively. In addition, let us assign an observed distribution of 100, 50, 75 and 775 in the four subgroups YY, YN, NY and NN, respectively. The expected distribution can be calculated according to the prevalence of the feature X as follows:
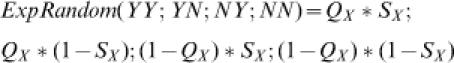
1


Where, *QX* (*SX*) is the fraction of proteins in the query (subject) dataset possessing the protein feature X.

According to the prevalence of the feature X in the query and subject protein sets, the expected distribution of 1000 protein pairs is 30, 120, 170, 680 in the four subgroups YY, YN, NY, NN.

Each of the four observed subgroups is comprised of randomly paired proteins, and pairs of homologs. Our objective is to estimate the amount of homologs among these 1000 protein pairs. Of course, the fraction of randomly paired proteins may vary between the four subgroups of the observed distribution. This noise distribution of randomly paired proteins, which resides hidden in the observed distribution, must comply with the relations between the expected distribution subgroups. Since both the noise and expected distributions are random distributions, the noise distribution is actually a smaller version of the expected distribution. Therefore, dividing the YY subgroup by the YN subgroup of the noise distribution, for example, should yield the same result that one would get by dividing the YY subgroup by the YN subgroup of the expected distribution.

Revealing the fraction of random protein pairs in one of the subgroups will enable the calculation of the fraction of random pairs in the remaining subgroups, according to the ratio of 30:120:170:680 between the expected subgroups. Towards this end, we first define the observed subgroup having the smallest observed-to-expected ratio (scaling subgroup), as consisting only of randomly paired proteins. This definition is likely to be incorrect, thus we overestimate the random pairs in this subgroup. In our hypothetical example, subgroup YN is selected for having the smallest ratio (50/120<75/170<775/680<100/30). Hence, we define all 50 protein pairs in the YN subgroup to be random ones. In practice, the scaling subgroup would typically be either the YN or the NY subgroups, as most homologs reside in the NN and YY subgroups, which are therefore usually larger than expected in random. Using the scaling subgroup, and the ratio between the four subgroups in the expected distribution (30∶120∶170∶680), it is possible to estimate the number of random pairs in the remaining three subgroups. The number of random pairs in the observed YY subgroup is 30*(50/120) = 12.5, in the NY subgroup it is 170*(50/120) = 70.8, and in the NN subgroup it is 680*(50/120) = 283.3. Next, dividing the sum of random pairs by the total number of protein pairs yields the total fraction of random pairs; its complementary fraction is our estimate of the fraction of homologs of the whole set, *Fhom*, as shown in Eq. (2) and (3). In our example,
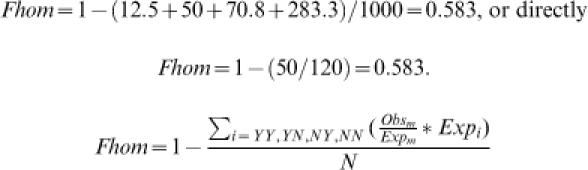
2

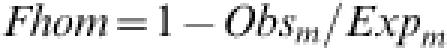
3Where, *Exp_i_* is the number of protein pairs in subgroup *i* according to the expected distribution, *Exp_m_* and *Obs_m_* are the expected and observed number of pairs in the subgroup having the smallest observed-to-expected ratio (scaling subgroup), and *N* is the total number of protein pairs.

The assumption that all pairs in one of the subgroups are random ones is strict, and the actual fraction of random pairs is typically lower. Thus, this method yields an underestimation of the real number of homologous protein pairs. It is important to note that the fraction of random protein pairs of the whole set cannot be larger. If this were the case, the scaling subgroup would have to be larger than it is. This is in order to comply with the larger fraction of random protein pairs. Hence, the fraction of random protein pairs is bounded by the size of this subgroup.

Finally, since we assume that homologous proteins perfectly conserve the presence of the protein feature X, the ratio between the YN and NY subgroups in the observed distribution, should be equal to the ratio in the expected distribution. Practically, as illustrated in our hypothetical example, an observed distribution may display a ratio that differs from the expected ratio (50/75 for the observed, and 120/170 for the expected). This indicates that the assumption of perfect conservation is not accurate. However, pairs of homologous proteins in which the protein feature was not conserved may only lead to further underestimation of *Fhom*, but never leads to an overestimation of *Fhom*. In summary, we present a method that provides an underestimation of the fraction of homologs in a set of protein pairs. The next sections describe implementation of this method, and its verification.

### Signal peptide as a feature suitable for *Fhom* estimation

The signal peptide is an abundant protein feature, encompassing about 20% of the human proteome. The signal peptide is a 15 to 40 long amino acid sequence, recognized by the signal recognition particles (SRP). The SRP leads the nascent protein to the endoplasmic reticulum, where the signal peptide is cleaved and removed. The new protein is then secreted from the cell or becomes transmembranal. SignalP is a reliable state-of-the-art tool for detection of signal peptides [17], [18]. Hence, the prevalence of signal peptides in the proteome of each organism in our analyses was determined by running the SignalP predictor on the curated RefSeq proteins of the organism. The results revealed that the signal peptide prevalence in the proteomes of human, mouse (*Mus musculus*), fruit fly (*Drosophila melanogaster*), and *Caenorhabditis elegans* is about 0.22, 0.22, 0.19 and 0.19, respectively.

**Figure 1:**
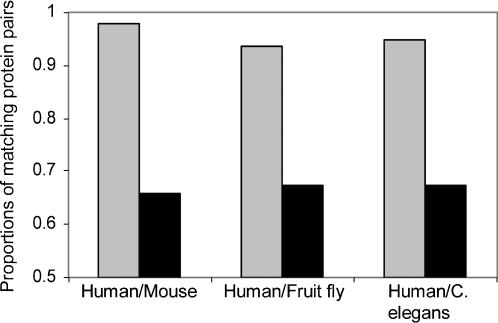
Signal peptide is a conserved feature of homologous proteins. Homologous proteins of human/mouse, human/fruit fly, and human/C. elegans were extracted from HomoloGene triplets. The proportion of matching protein pairs, in which both proteins have or lack a signal peptide, was calculated for each pair of organisms (gray columns). For comparison, the expected proportions (black columns), is calculated under the assumption that signal peptide is not conserved between homologs, using the prevalence of proteins having signal peptides in each organism.

We hypothesized that the presence of a signal peptide feature is conserved among homologous proteins. In order to test our hypothesis, sets of reliable homologous proteins were used as a standard of truth. We extracted human/mouse, human/fruit fly, and human/C. elegans homologous proteins from the HomoloGene triplets database. These organisms were selected to represent homology of different sequence divergence. In a random distribution, the expected proportion of protein pairs, in which both have or lack a signal peptide (YY+NN subgroups), is about 0.66, 0.67, and 0.67 for human/mouse, human/fruit fly, and human/C. elegans homologs, respectively (Figure 1, black columns). The observed proportion is clearly enriched in pairs of proteins that have the same signal peptide prediction (Figure 1, gray columns). Hence, the signal peptide feature is suitable for our estimation method. If homologous proteins would always have the same SignalP prediction, we expect the proportion to be 1. The actual value is below 1, meaning that not all homologous proteins have the same SignalP prediction. One explanation is that the SignalP tool is not perfect [17]. Hence, it is possible for some homologous proteins, although both have or lack a signal peptide, to be assigned opposite predictions. Another reason why the proportion is smaller than 1, could be the fact that not all homologous proteins have identical topology, or are secreted in non-classical pathways [19]. Nevertheless, there is a clear tendency for homologs to conserve the signal peptide.

**Figure 2:**
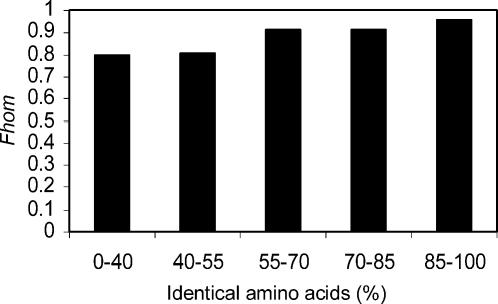
Alignment independence of the *Fhom Estimator.* HomoloGene homologous protein pairs were divided into five groups according to the identity level of their alignment. The organisms used are human, mouse, fruit fly and C. elegans. The figure reveals that the fraction of homologs estimation (Fhom) is applicable for both closely-related and distantly-related protein pairs.

One could argue that the reason SignalP assigns similar predictions to homologous proteins is because those proteins are very close in sequence; hence, SignalP, which uses the sequence as an input, will have similar predictions. To examine whether the signal peptide feature allows our method to be sequence alignment independent, HomoloGene protein pairs of the four selected organisms were divided into five groups according to their alignment identity, and the *Fhom* was estimated for each identity group (Figure 2). All of the groups comprise over 1000 protein pairs. Had our method been dependent on the alignment score values, one could expect a strong decrease in the homology estimates as a function of the decrease in the identity level. As revealed in Figure 2 this is not the case: our method estimates high values of homology (∼80%) even for the group of diverged homologous proteins that share less than 40% of their amino acid residues. In other words, the alignment itself has little effect on the homology estimation. Therefore, the method can be considered alignment independent, and the use of the signal peptide feature is applicable even where sequence alignment deteriorates considerably.

### Robustness of the *Fhom Estimator*

**Figure 3:**
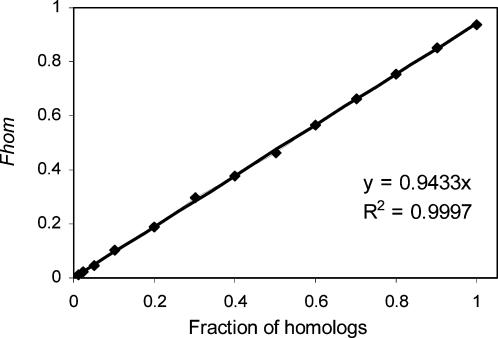
Robustness of the *Fhom Estimator.* *Fhom* was estimated to be 94% for HomoloGene human/mouse pairs. Next, random protein pairs were added according to the original signal peptide prevalence of these organisms. A linear regression curve and the coefficient of determination (R^2^) confirm that the *Fhom Estimator* has a wide dynamic range, and is robust to variation in signal-to-noise ratio.

In order to test the dynamic range of *Fhom*, we estimated the HomoloGene Human/Mouse *Fhom* for a start. Next, we artificially added random protein pairs, while maintaining the original prevalence of signal peptides. Figure 3 revealed that *Fhom* estimates well the real fraction of homologs for fractions between 0.01 and 1. The regression curve supports that the estimator always underestimates the real fraction of homologs, and that the *Fhom Estimator* has a wide dynamic range of homology.

### Sample size effect

**Figure 4:**
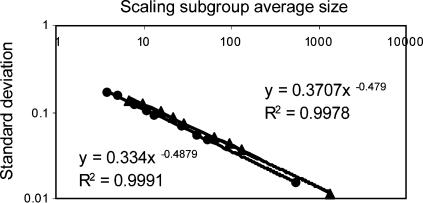
Size effect of scaling subgroup on the *Fhom Estimator* accuracy. Two sets of protein pairs of fruit fly and human Swiss-Prot proteins were estimated to have *Fhom* of 52% and 10%. Both alignment sets were used separately for the sample size simulation. Each set of pairs was divided into the observed subgroups. The proportions of the observed subgroups were used for simulating observed distributions of 9 different sample sizes ranging from 10,000 to 75. Each simulation was repeated 1000 times, and the average *Fhom, Fhom* standard deviation, and the average size of the scaling subgroup were taken. The power regression curves of the 52% homologs (lower left curve) and the 10% homologs (upper right curve) describe the correlation between the mean size of the scaling subgroup and the standard deviation of *Fhom*, with high coefficients of determination.

The accuracy of the method can be impaired when small sets of protein alignments are being assessed. To evaluate the sample size effect on the accuracy of the Fhom Estimator, we aligned the Swiss-Prot protein sequences of fruit fly and human using Blastp, with expectation value (*E-Value*)<100 cutoff. Signal peptides were removed from the protein sequences prior to the alignment. The results were sorted by their *E-Value*, and divided into 10 equal sized subsets of ∼54,400 pairs. A subset that had *E-Value*s between 0.052 and 1.7 was chosen arbitrarily, divided into the four subgroups, and was estimated to comprise 52% homologs. Next, we used the proportions of these observed subgroups to simulate the observed distributions for 9 different sample sizes ranging from 10,000 to 75. Each simulation was repeated 1000 times. For each sample size, the average *Fhom*, the *Fhom* standard deviation, and the average size of the scaling subgroup were calculated. To further validate the results, more subgroups of fruit fly/human alignments, having *Fhom* estimation between 83% and 10%, were used as a model for additional simulation. The size of the scaling subgroup directly affects the susceptibility of the method to statistical noise. It is more informative than the total sample size, which ignores possible differences in the protein feature prevalence. Figure 4 shows two simulation results for two sets of protein pairs having 52% *Fhom* and 10% *Fhom*, and their power regression curves. In both curves the standard deviation behaves like an N^−½^ function, where N is the size of the scaling subgroup. However, as the *Fhom* of the subgroup decreases, the coefficient multiplying the N^−½^ increases. Thus, to be on the safe side, it is recommended to make an upper bound to the standard deviation estimation by using the formula derived from a subgroup with very low *Fhom*:
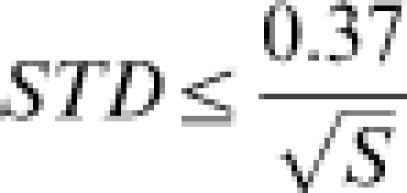
4Where *S* is the size of the scaling subgroup, and *STD* is the standard deviation. If *STD* of less than 0.05 is required, the scaling subgroup should be larger than 55. If *STD* may be as large as 0.1, a scaling subgroup as small as 14 is enough.

## Discussion

We have developed a computational method for estimating the fraction of homologs in a set of protein pairs. The method is simple and robust. The method is based on the assumption that homologous proteins either both have or lack a protein feature. For proteome-wide analyses, the presence of signal peptide, which tends to be conserved among homologs (Figure 1), was found to be a feature suitable for the method (Figure 2). For more focused analyses (e.g. sub-clustering of a protein family) a different feature may be more suitable.

For each pairwise alignment, sequence alignment methods calculate the *E-Value*, i.e. the number of different alignments that are expected to occur in a database search by chance, with scores equivalent to or better than the score of that specific alignment. This is an important measure, and indeed, alignments of most closely-related protein sequences have very low *E-Value*s. This way, it is possible to distinguish between closely-related homologous proteins and other protein pairs.

When looking for distantly-related protein sequences, a relatively high threshold on the *E-Value* should be used. This, of course, adds noise to the results, i.e. randomly paired proteins will mistakenly be recognized as homologs. The *Fhom Estimator*, which is independent of the alignment itself, enables estimating the amount of noise added, and is therefore beneficial for the *E-Value* threshold fine tuning. Furthermore, we found that the *Fhom Estimator* works well in cases of high as well as low signal to noise ratio (Figure 3).

An alignment *E-Value* depends on database size and composition, so it is impractical to define a universal cutoff, above which most data consists of random alignment pairs. The *Fhom Estimator* helps determine a specific *E-Value* cutoff for a particular set of alignment hits, above which the number of homologs is negligible. Therefore, the *Fhom Estimator* may be beneficial to assess sequence and structural alignment methods and fine tune their parameters.

In Kim & Kliger [16], we used the *Fhom Estimator* for the verification of a new scoring function (CTS) that facilitates the identification of viral piracy events [20]. A set of viral/human protein sequence alignments having low identity levels (10% to 35%) was divided into two subsets according to their CTS scores. The *Fhom Estimator* revealed that the subset of high CTS scores was enriched in homologs in comparison with the low-CTS-score subset.

In estimating *Fhom*, Eq. (1) is used for calculating the expected random distribution. The calculation is based on the fraction of proteins in the query and subject datasets possessing the protein feature X. It should be verified that the homology search method in use pairs non homologs as expected by chance, and not in a bias manner. In Kim & Kliger [16] such a verification was performed for Blastp. Proteins of dsDNA viruses were paired with human proteins using Blastp. A subset of pairs having an *E value*>5, which is considered statistically insignificant homology, was used as the set of non homologs protein pairs. We verified that Blastp pairing follows the expected random distribution, by comparing the fraction of pairs having the same signal peptide prediction (YY+NN subgroups divided by the total number of pairs) in the E value>5 subset, to the fraction expected by chance. Indeed, those fractions were similar. Hence, random pairing by Blastp, did not introduce a significant bias to the expected random distribution. Since we confirmed in Kim & Kliger that there is no bias in non homologs pairing by Blastp, we did not repeat this verification procedure in the current study. In general, to avoid random pairing bias, we recommend removing the protein feature sequences from the proteins' sequences, before executing the homology search. In our studies, we discarded the signal peptide sequences before running Blastp between the protein sets.

It is noteworthy that paralogs and orthologs are handled differently. Orthologous proteins are generally believed to have the same function in different organisms. Therefore, it is quite reasonable to assume that orthologous proteins will either both have or lack a signal peptide. However, the situation is more complex for paralogs. For example, there are intracellular and extracellular proteases that probably derived from the same protein ancestor. Therefore, choosing the presence of signal peptide as the feature for *Fhom* estimation might be problematic when dealing with distantly related paralogs. In summary, matching the right feature to the data is a crucial step for obtaining reliable results. In this study, pairs of paralogs had only a minute effect of further underestimating *Fhom* (data not shown), hence, they were included in all analyses.

An additional analysis of RefSeq protein sequences was performed. RefSeq proteins can be divided to manually inspected proteins and merely predicted proteins, which are assigned ‘NP_’ and ‘XP_’ accession prefixes, respectively. We found that the *Fhom* estimate of human/mouse XP/XP homologs (57%) is much lower than the *Fhom* estimate of NP/NP homologs (94%). Thus, the reliability of XP proteins in human and mouse is questioned. While in newly sequenced genomes most proteins are predicted and were not yet curated, in well-curated genomes (e.g. human, mouse, fruit fly and C. elegans genomes) we assume that many of the remaining predicted XP proteins might not represent real proteins (e.g. false products of pseudo-genes or fused genes). For that reason, we used only NP proteins of well-curated genomes in this study.

In conclusion, the *Fhom Estimator* is a simple and beneficial method to analyze the results of protein alignments. It can be used in quality assessments of homology search methods, which may share any level of sequence similarity, as being commonly done in bioinformatic analysis of proteins.

## Methods

### Data preparation

*Homo sapiens* (human), *Mus musculus* (mouse), *Drosophila melanogaster* (fruit fly), and *Caenorhabditis elegans* curated reference protein sequences (RefSeq build 11) were downloaded from NCBI's ftp web site [21], [22].

Swissprot [9] protein sequences of human and fruit fly were downloaded from NCBI Blast databases ftp web site (ftp://ftp.ncbi.nih.gov/blast/db/) on 21-June-06.

### Defining Homologs

HomoloGene triplets database of NCBI (Build 43.1) was used as the standard of truth for homologous proteins. HomoloGene databases contain pairs of homologous proteins from studied eukaryotic organisms that have a complete sequenced genome. In HomoloGene triplets database, each pair belongs to a group of at least three homologous proteins, and is considered highly reliable [22].

### Signal peptide prediction

Predicting whether a protein has a signal peptide was performed using the SignalP 3.0 prediction tool [18]. SignalP consists of two predictors - a neural network (NN) based predictor, and a hidden Markov model (HMM) based predictor. Proteins having contradicting predictions assigned by the two predictors were discarded.
